# Sodium-Ion Battery at Low Temperature: Challenges and Strategies

**DOI:** 10.3390/nano14191604

**Published:** 2024-10-04

**Authors:** Yan Zhao, Zhen Zhang, Yalong Zheng, Yichao Luo, Xinyu Jiang, Yaru Wang, Zhoulu Wang, Yutong Wu, Yi Zhang, Xiang Liu, Baizeng Fang

**Affiliations:** 1 School of Energy Sciences and Engineering, Nanjing Tech University, Nanjing 211816, China; joyeah1016@163.com (Y.Z.); yalongyun@163.com (Y.Z.); ycluo2023@njtech.edu.cn (Y.L.); jiangxinyu@njtech.edu.cn (X.J.); 202361208036@njtech.edu.cn (Y.W.); yw19@njtech.edu.cn (Y.W.); zhangy@njtech.edu.cn (Y.Z.); iamxliu@njtech.edu.cn (X.L.); 2 School of Chemistry and Molecular Engineering, Nanjing Tech University, Nanjing 211816, China; zhangzhen202109@163.com; 3 School of Chemical Engineering and Energy Technology, Dongguan University of Technology, Dongguan 523808, China

**Keywords:** sodium-ion battery, low-temperature performance, modification strategy

## Abstract

Sodium-ion batteries (SIBs) have garnered significant interest due to their potential as viable alternatives to conventional lithium-ion batteries (LIBs), particularly in environments where low-temperature (LT) performance is crucial. This paper provides a comprehensive review of current research on LT SIBs, focusing on electrode materials, electrolytes, and operational challenges specific to sub-zero conditions. Recent advancements in electrode materials, such as carbon-based materials and titanium-based materials, are discussed for their ability to enhance ion diffusion kinetics and overall battery performance at colder temperatures. The critical role of electrolyte formulation in maintaining battery efficiency and stability under extreme cold is highlighted, alongside strategies to mitigate capacity loss and cycle degradation. Future research directions underscore the need for further improvements in energy density and durability and scalable manufacturing processes to facilitate commercial adoption. Overall, LT SIBs represent a promising frontier in energy storage technology, with ongoing efforts aimed at overcoming technical barriers to enable widespread deployment in cold-climate applications and beyond.

## 1. Introduction

Lithium-ion batteries (LIBs) have many advantages, such as high energy density, long life cycle, etc., so LIBs are widely used in portable electronics, electric vehicles, and the aerospace industry. However, the global shortage of lithium resources and uneven geographical distribution have increased the cost of LIBs and limited their application. Sodium and lithium belong to group IA elements and have similar physicochemical properties. As sodium resources are abundant and widely distributed, sodium-ion batteries (SIBs) are expected to become a promising next-generation energy storage system.

An electrochemical cell has two electrodes: the anode and the cathode, separated by an electrolyte. The electrolyte can be a liquid or solid. Solid electrolytes can be used with gas or liquid electrodes; they can also work with solid electrodes, but solid–solid interfaces can be tricky unless the solid electrolyte is a polymer or the solid electrode is very thin. Solid electrodes separated by a liquid electrolyte are divided by a permeable barrier [1]. Similar to LIBs, SIBs follow a comparable mechanism, the “rocking chair mechanism” [2], whereby ions shuttle reversibly between two electrodes, conducting ions in the electrolyte. Typically, during the charge–discharge process of batteries, challenges are involved in terms of operating safety, high reactivity, Na dendritic growth, and significant volume changes, which pose a considerable obstacle to their stable operation [3,4]. Room-temperature (RT) SIBs containing anodes of metal compounds and flammable organic solvent electrolytes have serious safety problems. However, introducing non-flammable, highly fluorinated structures as bridge solvents has enabled superior miscibility into fluorinated carbonate electrolytes [5]. The dendrite growth that causes electrical shorting and cell failure is mainly due to the cycling instability of the solid electrolyte interphase (SEI) layer of the metal anode [6,7]. Thus, many comprehensive strategies in electrolyte formulation, artificial single event latch-up engineering, current collector design, etc., have been implemented to stabilize Na metal and permit its long-term operation at RT. However, commercial batteries in low temperatures (LTs) (usually referring to below 0 °C, often between −20 °C and −40 °C) cannot work well. Even at 0 °C, electric vehicles often have a shorter range. When temperatures drop below freezing, the batteries’ capacity, voltage, power, and lifespan are greatly reduced [8]. The LT performance of SIBs remains to be further studied and summarized. Developing the performance of SIBs at LT is crucial for several reasons. First, this can expand the application of energy storage in colder climates, ensuring reliable performance in regions where traditional batteries may falter. Second, compared to Earth’s geologically moderate environments, extraterrestrial l celestial bodies present a spectrum of extreme conditions. The absence of a substantial atmosphere on celestial bodies such as Mars and the moon eliminates atmospheric regulation, leading to significant temperature fluctuations. It is important to advance the battery industry’s capabilities in extreme terrestrial conditions for space work [9,10]. Additionally, enhancing performance at LTs can support electric vehicles and grid storage solutions, ultimately improving energy efficiency and reducing reliance on fossil fuels [11,12].

Up to now, research on improving the LT performance of SIBs has mainly focused on modifying electrode materials. These improvements aim to increase ion diffusion rates, enhance electronic conductivity, and ensure cycle stability. Additionally, efforts are being made to optimize electrolytes to lower viscosity and improve interface compatibility. Specifically, for anode materials, carbon-based materials can accelerate Na^+^ insertion and diffusion in graphite-like domains by expanding the interlayer space. Doping with heteroatoms and surface coatings can provide more active sites for adsorption, while optimizing porous structures allows for sodium cluster storage in suitable pores [13,14]. For alloy metal materials, the focus is on surface coating strategies and metal compounds to reduce volume changes and increase Na^+^ storage. Metal sodium can be used to create artificial SEI and modify electrolytes, helping to reduce unstable dendrite growth and improve SEI stability. For cathode materials, polyanionic compounds (PACs) exhibit excellent conductivity through carbon coating, micro-nano structures, and element doping [15,16]. Prussian blue analogs (PBAs) can increase sodium content by introducing transition metals. Layered transition metal oxides (TMOs) focus on coordinating Na^+^ and doping with various transition metals to increase Na^+^ content. In terms of electrolytes, adjusting solvent components, selecting the best sodium salts, and using additives are common methods to lower electrolyte viscosity, stabilize the SEI, and reduce Na^+^ dissolution energy [17]. Current research is building on previous studies to explore and innovate further. This review follows a similar logical structure, allowing us to compare current research directions with past findings. However, there is still a long way to go, and the path of innovation awaits many researchers to pursue this avenue together.

This review discusses the latest research progress of SIBs at LTs, including electrode materials and electrolytes. In terms of electrodes, anodes can be classified as carbon-based anodes, titanium-based anodes, and conversion and alloy-based materials. The cathode can include layered transition metal oxides (TMOs), polyanionic compounds, Prussian blue analogs, organic polymers, and others. At the same time, corresponding strategies to improve the performance of the electrode are summarized. In the third part, the merits and demerits of ether-based and carbonate-based electrolytes are compared to demonstrate their potential and limitations, thus providing application principles for ether-based and carbonate-based electrolytes at LTs to maximize their advantages. Furthermore, mitigation strategies for LT electrolytes are emphasized to guide future electrolyte design.

## 2. Research Progress of SIBs in LT Environment

The optimal operating temperatures of LIBs are all above 0 °C. Some studies indicate that the rate performance and life cycle of LIBs significantly decrease in LT environments [8]. Internal battery resistance increases drastically at extreme conditions below −20 °C, which inevitably leads to a considerable decrease in power sourcing/sinking capabilities [18,19]. The main factors restricting the performance of LT LIBs are as follows: (1) at LTs, the viscosity of the electrolyte increases and ionic conductivity significantly decreases, even leading to electrolyte freezing at −30 °C, which hinders the shuttling of lithium ions between the anode and cathode; (2) the internal resistance increases during charging, slowing down the lithium-ion insertion kinetics, making lithium plating, which can cause side reactions with the electrolyte, leading to thickening of the interfacial layer at the electrode/electrolyte interface and potentially forming large lithium dendrites or “dead lithium” on the anode surface, resulting in internal short circuits and thermal runaway safety issues; and (3) the lithium-ion de/insertion process is limited, reducing Coulombic efficiency, complicating charging and discharging, and further decreasing the lifespan of LIBs. But for SIBs, when working at temperatures lower than 0 °C, problems occur such as a rapid decline in the discharge voltage plateau, low discharge capacity, poor rate performance, and dendrite growth, which seriously decrease the life cycle of the batteries, SIBs work relatively stably at LTs and maintain higher efficiency than LIBs. Therefore, in recent years, more and more research has been conducted on the LT performance of SIBs. But the mechanism of LT failure of SIBs has not been completely clarified, especially since research on electrode materials and electrolytes has not yet formed a system. This review summarizes the problems and challenges faced by SIBs. In addition, the research progress on the three aspects of the cathode, anode, and electrolyte of LT SIBs is summarized, respectively. Finally, the future research direction is envisioned. This review provides a reference for developing excellent-performance LT SIBs in the future.

### 2.1. Anode

SIBs have a similar energy storage mechanism to LIBs, and anode materials can be categorized into intercalation materials [20], conversion-type materials [21,22], and alloy-based materials [23,24]. Intercalation materials can store and release energy by inserting and extracting sodium ions. These materials usually have a layered structure, allowing them to hold sodium ions between their layers, which leads to efficient electrochemical reactions. For example, hard carbon is a typical intercalation material, and its use in SIBs has already been commercialized, showing great promise for the future. Unlike intercalation materials, conversion materials undergo phase decomposition while absorbing and releasing sodium, which involves bond breaking and new bond formation. Like lithium, the conversion reaction with sodium can be written in a specific formula: M_a_X_b_ + (b · z) Na ↔ aM + bNa_z_X. For typical conversion materials, M represents transition metal elements like Fe, Co, Ni, Cu, Mn, etc.; X stands for nonmetal elements including O, N, F, S, Se, P, H, etc. These M_a_X_b_ materials have higher theoretical capacity than pure M metals. On the other hand, conversion electrodes often turn into nanoparticles during charge and discharge due to phase decomposition. Metal M can form amorphous or crystalline nanoparticles, while nucleated Na_z_X tends to surround M nanoparticles. Ideally, M nanoparticles and the Na_z_X matrix form a dual continuous conductive network, which helps in transferring electrons and Na^+^ and makes the conversion reaction reversible. The interface between the conductive M phase and the ionic conductive Na_z_X can store extra sodium through a “job-sharing” mechanism, leading to increased capacity [25,26]. Group 14 and 15 elements, which include metals like Sn, Pb, and Bi, metalloids such as Si, Ge, As, and Sb, and the polyatomic nonmetal P, are known to create binary compounds with Na. These electrode materials, which either form alloys with Na or Na binary compounds, have been explored as potential negative electrodes for rechargeable SIBs [27,28].

Intercalation electrodes exhibit excellent reaction kinetics but lower energy density. In this part, we discuss the most representative members of them: carbon-based materials [29,30,31] and titanium-based materials [32,33]. Conversion reaction electrodes also provide high capacity and good LT performance. Still, the sodium storage mechanism based on the pseudocapacitive effect leads to high self-discharge and complex working mechanisms, making them difficult to apply in practice. Alloy-type electrodes offer better LT performance and energy density, but their cycling life is poorer due to big volume changes during cycling. Organic materials, due to their flexible structure, can provide high sodium ion migration rates, but the large dissolution of organic solvents during cycling leads to serious capacity decay. Therefore, organic materials are not suitable for use in SIBs at LTs. Currently, new negative electrode materials with high capacity are being developed. Reducing particle size, doping, modifying the solid–liquid interface, and designing electrode morphology are common strategies to improve cycling life, reduce diffusion paths in the ion-solid phase, enhance material ion conductivity, and promote charge transfer processes.

#### 2.1.1. Carbon-Based Materials

Carbon-based materials are the most studied anodes for NIBs, due to their natural abundance and renewability, environmentally friendly properties, low cost, and stability, and some innovative research has also been carried out on their preparation [34]. Graphite as a commercial anode in LIBs is thermodynamically inaccessible for Na, while delivering a lower storage capacity (about half of that of LIBs) with the binding of solvated Na ions. Earlier, reduced graphene oxide (rGO) was also studied for LT SIBs [35]. However, there is less research on using it as the main electrode material. It is often used as a carbon material for doping, which helps improve the conductivity of different electrode materials. Nongraphite hard carbon (HC) is the potential anode material for SIBs due to its high reversible capacity (≈300 mAh g^−1^) and long low-potential charge/discharge plateaus (<0.1 V) [36], and it provides a facile and stable accommodation of Na^+^ compared with graphite [37,38]. The large polarization attributed to sluggish kinetics is an obstacle for HC for LT SIBs. Therefore, strategies such as introducing defects on the surface and creating abundant active sites are introduced to improve the electrochemical performance of carbon-based anode materials [39].

**Figure 1:**
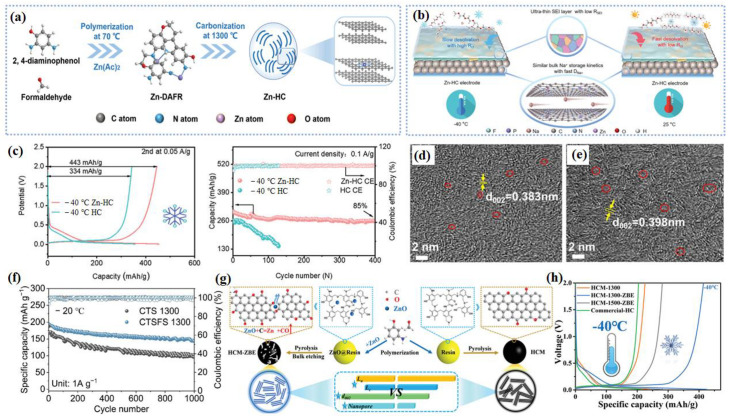
(**a**) A schematic illustration of the synthesis for Zn-HC. (**b**) A schematic diagram of the enhanced rate capability mechanism. (**c**) The low-temperature performance (−40 °C) of the GCD curves (the second cycle) and cycling stabilities. Reproduced with permission from ref. [40]. Copyright 2023 Wiley-VCH GmbH. HRTEM image of (**d**) CTS 1300, (**e**) CTSFS 1300. (**f**) Cycling performances of CTS 1300 and CTSFS 1300 at −20 °C. Reproduced with permission from ref. [41]. Copyright 2024 Elsevier. (**g**) A schematic diagram illustrating the synthesis route of the samples. (**h**) The GCD curves at −40 °C (the second cycle). Reproduced with permission from ref. [42]. Copyright 2023 Wiley-VCH GmbH.

For example, as shown in Figure 1a, a hard carbon with atomically incorporated zinc single atoms (Zn-HC) to modulate the bulk and surface structure was reported. Lu et al. confirm that thanks to the ultrathin NaF polymeric SEI layers contributed by interfacial catalysis of the Zn-N_4_-C configuration and the in situ induced local electric field (LEF) by the Zn-N_4_-C configuration, both the bulk and interfacial Na^+^ storage kinetics are enhanced significantly, leading to the unprecedented rate capability and initial columbic efficiency (ICE) of the Zn-HC electrode (Figure 1b). Additionally, the moderate adsorption energy and robust inorganic-rich SEI can suppress the formation of Na dendrites, thus enabling the long cycle performance in the LT environment [40]. The LT performance of the samples was tested at an extreme LT (−40 °C) to simulate the actual application scenario. A remarkably high charge capacity of 443 mAh g^−1^ is achieved for Zn-HC at a 0.05 A g^−1^ current density. At the same time, it has an extremely high reversible capacity (546 mAh g^−1^) and excellent ICE (84%) at −40 °C (Figure 1c). To synthesize hard carbon (CTSFS 1300) with smaller particle sizes, larger interlayer spacing, and larger closed pores, Yuan used flash Joule heating (FJH) [41]. For example, Figure 1d,e show that CTSFS 1300 had a larger interlayer spacing of 0.398 nm (the yellow arrow) than CTS 1300 (which is carbonized by slow heating). Benefiting from the ideal structure, CTSFS 1300 has excellent LT performance (Figure 1f). More importantly, Yin et al. found that the carbon p-band center upholds a linear relationship with both the Na^+^ adsorption energy (Ea) and diffusion energy barrier (Eb), which can be readily manipulated by adjusting the physical parameters of the hard carbons (HCs) [42]. They synthesized HC microspheres with a well-regulated microstructure via the ZnO-assisted bulk etching method (Figure 1g). As shown in Figure 1h, this material delivers an incredible reversible capacity at LTs (426 mAh g^−1^ @ −40 °C at 0.05 A g^−1^). Ultimately, carbon-based anode materials, such as graphite and nongraphite hard carbon (HC), show promise in SIBs. Graphite, though limited by low storage capacity for sodium, remains stable in LIBs. In contrast, HC offers a higher capacity and better sodium ion accommodation. However, HC faces challenges like sluggish kinetics. Strategies like defect introduction improve its performance. For instance, atomically incorporating zinc single atoms into HC enhances its kinetics and long-term stability, vital for LT applications. Innovative synthesis methods, like flash Joule heating, further optimize HC’s structure for improved LT performance, showing significant potential for future battery technologies. The application of hard carbon in SIBs at LTs is still worth exploring.

#### 2.1.2. Titanium-Based Materials

Titanates have been widely reported as anode materials for SIBs. These titanium-based materials can be divided into several categories, including layered Na/Ti-containing oxides, MXene, TiO_2_ poly morphs, etc. Due to the various crystalline phases, significant structural stability, and high abundance, titanium-based oxides exhibit considerable sodium storage potential at LTs [43,44]. Additionally, their low volume expansion (<4%) during discharge/charge according to typical insertion mechanisms enhances their suitability for SIBs [33,45,46].

**Figure 2:**
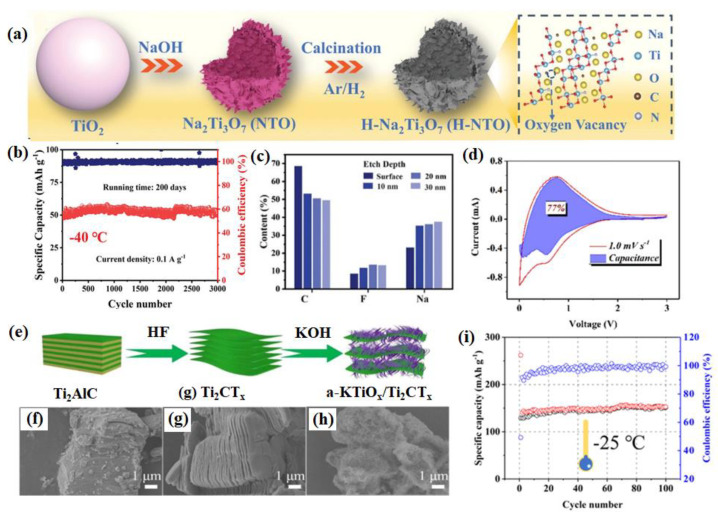
(**a**) Schematic diagram of synthetic route for H-NTO microspheres. (**b**) Cycle performance at −40 °C of H-NTO electrode. (**c**) Atomic ratio of C, F, and Na in SEI formed for cycled H-NTO electrode. Reproduced with permission from ref. [47]. Copyright 2023 Wiley-VCH GmbH. (**d**) Capacitive contributions to charge storage at 1.0 mV s^−1^ of a-KTiO_x_/Ti_2_CT_x_. (**e**) Schematic illustration for preparation of a-KTiO_x_/Ti_2_CT_x_. FESEM images of (**f**) pristine Ti_2_AlC MAX, (**g**) multi-layered Ti_2_CT_x_, and (**h**) a-KTiO_x_/Ti_2_CT_x_. (**i**) Cycling performances at 0.1 A g^−1^ of a-KTiO_x_Ti_2_CT_x_ at −25 °C. Reproduced with permission from ref. [49]. Copyright 2022 Elsevier Inc.

**Figure 3:**
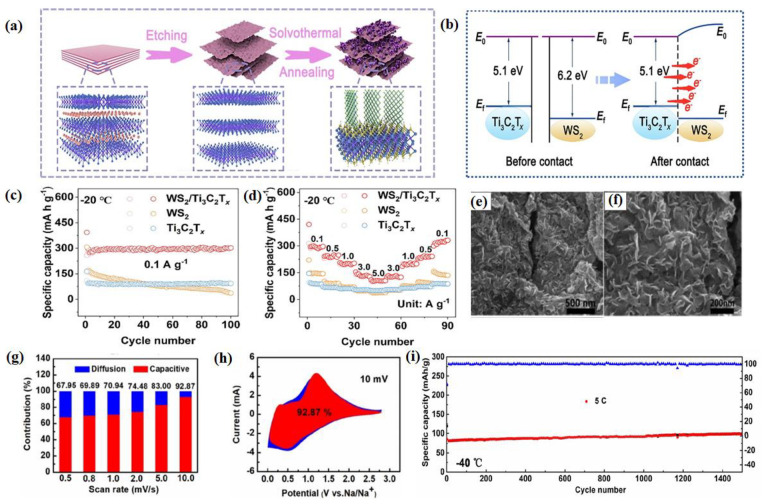
(**a**) Illustration of fabrication of WS_2_/Ti_3_C_2_T_x_ heterojunction. (**b**) Illustration of electron redistribution at interface between layered WS_2_ and Ti_3_C_2_T_x_ MXene. (**c**) Sodium storage performance at −20 °C: cycling at 0.1 A g^−1^ of WS_2_/Ti_3_C_2_T_x_ heterojunction, pure WS_2_, and Ti_3_C_2_T_x_ MXene. (**d**) Rate capabilities of WS_2_/Ti_3_C_2_T_x_, pure WS_2_, and Ti_3_C_2_T_x_ MXene. Reproduced with permission from ref. [50]. Copyright 2023 Science Press and Dalian Institute of Chemical Physics, Chinese Academy of Sciences. Published by ELSEVIER B.V. and Science Press. (**e**,**f**) SEM images of in situ TiO_2_@rGO. (**g**) Contribution ratio of capacitive and diffusion-controlled charges in TiO_2_@rGO at different scan rates. (**h**) Separation of capacitive (red region) and diffusion currents in TiO_2_@rGO at a scan rate of 10 mV S^−1^. (**i**) Charge and discharge capacity of TiO_2_@rGO versus cycle number at current densities of 5C in temperature of −40 °C. Reproduced with permission from ref. [51]. Copyright 2020 Elsevier B.V.

A novel material with a hollow Na_2_Ti_3_O_7_ microsphere (H-NTO) with oxygen defects (Figure 2a) was reported, and it has a unique chemical bonding NTO/C(N) interface [47]. The optimized electrolyte allows the H-NTO electrode to cycle stably for 200 calendar days at −40 °C without capacity degradation (Figure 2b). The excellent cycling stability is attributed to the NTO/C(N) interface and the stable SEI formed by the highly adaptive electrolyte/electrode interface. More specifically, Figure 2c shows that the contents of C and F decrease and increase with etching depth, respectively, indicating the alternating presence of organic–inorganic boundary layers in the SEI layer. This integrated organic–inorganic boundary layer is the most stable in rechargeable batteries. A tunnel-like structure Na_2_Ti_6_O_13_@C nanowire was synthesized and utilized as the anode material for LT SIBs [48]. The authors discovered that the surface carbon has a positive effect on the electrochemical behavior of a Na_2_Ti_6_O_13_ electrode by improving the sodium ions’ diffusion coefficient at low working temperatures. As a result, in the full-cell electrochemical performance, the Na_2_Ti_6_O_13_@C||NVP remained at nearly 60% capacity at lower temperatures of −20 °C compared to that at 20 °C, and an acceptable rate capability was discovered in the wide-temperature range from 20 °C to −40 °C without the special LT electrolyte. Li et al. achieved the in situ growth of amorphous potassium titanium oxide (KTiO_x_) nanoribbons (a-KTiO_x_) on multilayer Ti_2_CT_x_ MXene, forming a three-dimensional (3D) interconnected heterostructure (a-KTiO_x_/Ti_2_CT_x_) [49] (Figure 2e–h). The novel combination of one-dimensional (1D) amorphous nanoribbons with two-dimensional conductive MXene nanosheets results in low mechanical strain within the heterostructure during ion insertion/deinsertion processes, rapid ion diffusion kinetics, low activation energy barriers at interfaces, and high capacitance contributions to charge storage (Figure 2d), thereby exhibiting strong sodium storage performance at LTs. As shown in Figure 2g, the stable cycling performance of a-KTiO_x_/Ti_2_CT_x_ can still be maintained at −25 °C, along with a reversible capacity of 112.6 mAh g^−1^ after 100 cycles. The interconnected nanostructure of a WS_2_/Ti_3_C_2_T_x_ heterojunction with a built-in electric field (BIEF) was established by Li and his group [50], and this served as a model to reveal the positive effects of heterojunction design and a BIEF on altering reaction kinetics and electrochemical activity (Figure 3a), forming a BIEF and an “ionic reservoir” spontaneously at the heterogeneous interface (Figure 3b). The calculation results ensure rapid diffusion kinetics and good structural coupling. Therefore, the WS_2_/Ti_3_C_2_T_x_ heterojunction exhibits excellent LT sodium storage performance (293.5 mAh g^−1^ at 0.1 A g^−1^ after 100 cycles even at −20 °C) (Figure 3c). Meanwhile, the WS_2_/Ti_3_C_2_T_x_ electrode also exhibits an outstanding rate capability at −20 °C (Figure 3d), which further confirms its robust LT tolerance.

Deng and co-workers prepared oxide graphene-loaded titanium dioxide (TiO_2_) nanosheet composites (TiO_2_@rGO) prepared by an in situ method [51]. Previous first-principle calculations by Chen et al. showed that the intimate integration of graphene with TiO_2_ reduces the diffusion energy barrier and thus enhances the Na^+^ intercalation pseudocapacitive process [52]. The structure and morphology of the TiO_2_@rGO sample could be detected from the SEM, as shown in Figure 3e,f. As shown in Figure 3g, the TiO_2_@rGO electrode had a 67.9% capacitive contribution at a scan rate of 0.5 mV S^−1^. Moreover, when the scan rate reaches 10 mv s^−1^, the capacitance contribution can reach 92.8% (Figure 3h), which is quite a high level compared to the other one in their work. The enhanced pseudocapacitive process could increase the kinetic reaction of the cell, especially at high current densities, and produce an excellent electrochemical performance. At quite LTs (−40 °C), the capacity of this electrode can still reach 120 mAh g^−1^ after 1500 cycles at the current density of 5C (Figure 3i), one of the best-reported LT properties for SIBs until 2019. In conclusion, titanium-based materials are extensively studied as anodes for SIBs, and to improve their electrochemical performance, they are mixed with conductive C-based skeletons and heterojunction structures are widely used. Abundant research has been carried out to combine titanium-based materials with conductive carbon scaffolders, and satisfactory results have been achieved. In the future, should we start from the main body of the material, develop simpler preparation methods, or try to accelerate the realization of commercial applications from the perspective of industrial preparation of materials?

#### 2.1.3. Conversion and Alloy-Based Materials

**Figure 4:**
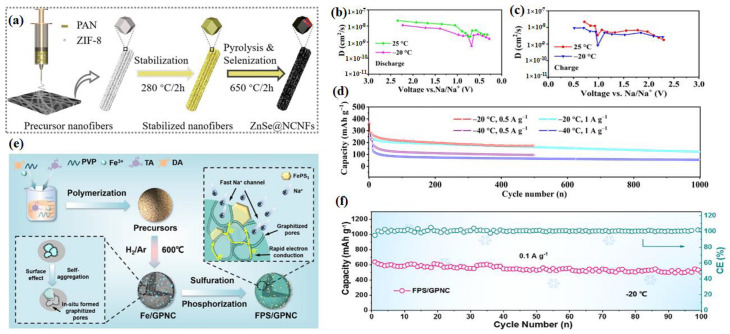
(**a**) Schematic diagram of the synthesis procedure of ZnSe@NCNFs. (**b**,**c**) Na^+^ diffusion coefficient during cycle process. (**d**) Electrochemical performance of ZnSe@NCNFs in low temperatures for SIBs on 0.5 and 1 A g^−1^. Reproduced with permission from ref. [54]. Copyright 2021 American Chemical Society. (**e**) Synthesis diagram of FPS/GPNC. (**f**) Long cycling stability of FPS/GPNC at −20 °C. Reproduced with permission from ref. [55]. Copyright 2022, American Chemical Society.

**Figure 5:**
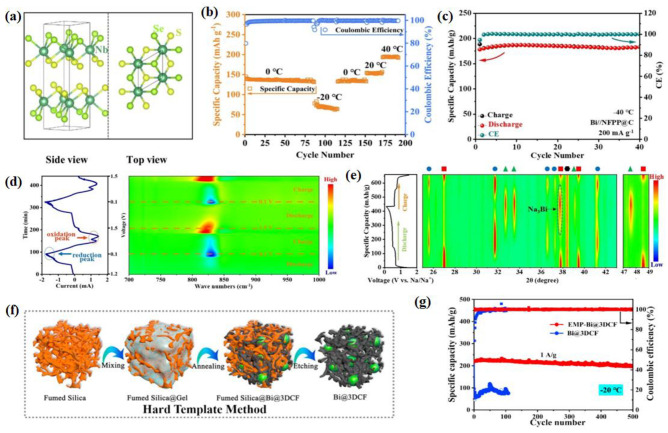
(**a**) The schematic of crystal structure for NbSSe. (**b**) Cycle performance for NbSSe at different temperatures (−20 °C, 0 °C, 20 °C, 40 °C). Reproduced with permission from ref. [60]. copyright 2022 Elsevier B.V. (**c**) Cycle stability of the Bi//NFPP@C batteries at −40 °C. (**d**) CV curve in current versus time form and corresponding 2D color-filled contour plot in wavenumber versus time form of the in situ FTIR/ATR test of the Na//Bi half battery. (**e**) The galvanostatic charge/discharge curves of Na//Bi half battery during the in situ XRD test and corresponding 2D color-filled contour plot. Reproduced with permission from ref. [67]. Copyright 2022 Wiley-VCH GmbH. (**f**) Schematic illustration of the synthesis process of Bi@3DCF. (**g**) cycling stability at 1 A g^−1^ for EMP-Bi@3DCF and Bi@3DCF at −20 °C. Reproduced with permission from ref. [65]. Copyright 2020 Elsevier Ltd.

The most common solution to simultaneously boost the structural stability and electrode kinetics of conversion-type anodes at LTs is utilizing conductive scaffolds for anode fabrication [53,54,55,56], which could increase electronic conductivity and ensure electrode integrity during conversion reactions. For example, Wang et al. reported a high Na^+^ diffusion coefficient (5.57 × 10^−10^~1.12 × 10^−8^ cm^2^ s^−1^) of a nitrogen-doped porous carbon nanofiber-encapsulated ZnSe nanoparticle (ZnSe@NCNFs) composite anode at −20 °C (Figure 4a–c). Its impressive electrochemical properties are primarily due to the peculiar porous framework and ultrafine ZnSe nanoparticles, as well as robust N-doped carbon matrix, which provides a penetrating network for fast ion/electron transfer and alleviates the pulverization of ZnSe during a sodiation/desodiation reaction [54]. In Figure 4d, it is found that the ZnSe@NCNFs electrode still maintains a reversible capacity of 122.8 mAh g^−1^ under 1.0 A g^−1^ at −20 °C after 1000 cycles. Even at −40 °C, ZnSe@NCNFs still provide capacities of 94.7 mAh g^−1^ at 0.5 A g^−1^. The LT practicability of the ZnSe@NCNFs electrode is all profit by the higher D_Na_^+^ and reasonable structure design. Huang et al. construct FePS_3_ submicron/micron particles embedded in an N-doped carbon framework (Figure 4e) with in situ-formed graphitized pores (FPS/GPNC). Such a unique graphitized pore architecture affords three-dimensional open interconnected channels for Na^+^ for fast transportation and forms a superior conductive network composed of graphitized pore walls to accelerate electron conduction [55]. Due to the reasons mentioned above, the LT performance (−20 °C) of FePS_3_ is obtained (502.6 mAh g^−1^ maintained after 100 cycles at 0.1 A g^−1^), indicating huge potential for practical applications (Figure 4f). Moreover, rGO [53,57,58], graphene [56], and MXene [59] are usually used as conductive scaffolds in the conversion-type anode. Unlike the strategies mentioned above, two-dimensional NbSSe nanoplates for SIBs were prepared by Zhou and his co-workers. The strategy of inducing interlayer anionic ligands in two-dimensional NbSSe nanoplates is employed to consolidate the interlayer band gap and optimize the electronic structure. It combines complementary benefits from two kinds of anionic ligands with high conductivity and good affinity with sodium ions at LTs [60]. Figure 5a shows that Nb atoms are sur-rounded by S or Se atoms on either side in an orderly manner, forming the sexangle together. So, Figure 5b exhibits a stable cycling performance at 0 °C during nearly 90 cycles at 0.2C. After by decreasing the temperature to −20 °C, the cycling performance is still acceptable. When returning to 0 °C, the reversible capacity nearly reaches 140 mAh g^−1^ and the coulombic efficiency is close to 100%. In conclusion, employing conductive scaffolds such as nitrogen-doped porous carbon nanofibers or in situ-formed graphitized pores significantly enhances the LT performance of conversion-type anodes. Exemplified by impressive results such as high Na^+^ diffusion coefficients and stable cycling capacities even at extreme temperatures like −40 °C, this innovative approach, coupled with robust structural designs, offers promising prospects for practical applications in next-generation SIBs, demonstrating superior electrochemical properties and stability under challenging conditions.

The problems concerned with alloy anodes are similar to conversion-type anodes at LTs, including huge volume variation during the alloying process and decreased kinetics. Research promoting the LT performance of alloy anodes is still at the primary stage, but some strategies have been proven effective in simultaneously stabilizing anode structures and boosting electrode kinetics, such as developing binary alloys [61,62], fabricating a robust SEI [63], and compositing with carbon materials [64,65,66], which may provide inspirations for future research in this field. Diglyme cointercalation with Na^+^ into a Bi anode was proposed to boost the charge transfer process at LTs [67]. This merit was claimed to support the high-rate performance of a Bi anode-based full battery at an extreme LT of −70 °C. As shown in Figure 5c, the Bi//NFPP@C battery shows excellent cycle stability with negligible capacity decay within 40 cycles at −40 °C. By using in situ Fourier transform infrared–attenuated total reflection (FTIR/ATR) spectroscopy analysis and XRD characterization, Li and his co-workers verified that solvated Na^+^ can be directly stored by the Bi anode through an alloying reaction without the de-solvent process, which is critical for LT operation (Figure 5d,e). Wang et al. [65] developed a preparation process in which Bi nanoparticles with a size of 40–50 nm were encapsulated in a 3D interconnected sponge-like N-doped carbon framework by combining the sol–gel method and fumed silica template (donated as Bi@3DCF) (Figure 5f), and the formation of the EMP-Bi@3DCF was achieved via electrochemical milling. The first step of the EMP is the fabrication of an electrochemical cell with a Bi@3DCF electrode versus a Na metal counter. The cell underwent galvanostatic charge/discharge between 0.1 and 1.5 V at room temperature for 10 cycles, and the obtained materials were noted as EMP-Bi@3DCF. The interconnected porous structure with both ionic and electronic transporting phases enables quite an efficient mix-conducting network, leading to an ultrafast charge/discharge rate even at LTs (−20 °C) (Figure 5g) [65]. Efforts to enhance the performance of alloy-type materials at LTs are promising but in the early stages. For instance, Bi anodes with diglyme cointercalation achieved notable charge transfer enhancement at −70 °C, ensuring high-rate performance in full batteries. Novel approaches such as Bi@3DCF composites further demonstrate efficient ionic and electronic conductivity, enabling rapid charge/discharge rates even at −20 °C.

### 2.2. Cathode

Currently, the cathode materials for SIBs mainly include polyanionic compounds [68,69], Prussian blue analogs [70], layered transition metal oxides (TMOs) [71,72,73], and organic polymers [74], among others. Firstly, polyanionic materials are widely researched due to their good structural stability and high ionic conductivity [75,76]. Meanwhile, Prussian blue and its analogs (PBAs) possess open three-dimensional frameworks conducive to sodium ion storage and diffusion, rendering them with significant potential as cathode materials for SIBs. Layered transition metal oxides offer advantages such as low cost, excellent LT performance, high thermal stability, good safety performance, high energy density, and environmental friendliness. Organic polymers have garnered interest as potential cathode materials for SIBs due to their lightweight nature, flexibility in design, and potentially lower cost compared to traditional inorganic materials. These polymers can be tailored for specific properties, such as high capacity and good cycling stability [77,78]. However, they often face challenges in achieving excellent LT performance. One key issue is their inherent molecular flexibility, which can lead to decreased ion transport kinetics at lower temperatures. This results in reduced efficiency and capacity retention when the battery operates in cold environments [79,80]. Additionally, organic polymers may suffer from increased electrode polarization and decreased conductivity at LT, further hindering their overall performance and practical usability in such conditions. Therefore, we will not discuss organic compound cathodes further in the following text.

#### 2.2.1. Polyanionic Compounds

In various materials used for SIBs, electrode materials based on sodium superionic conductors (NASICONs) are one of the most promising candidates for sodium storage electrodes due to their significant structural stability and high ionic conductivity [81]. The molecular formula of NASICONs can be represented as Na_x_MM’ (XO_4_)_3_ (where M or M’ = V, Fe, etc., X = P or S, x = 0–4). They feature corner-sharing MO_6_ (M’O_6_) octahedra and polyhedral XO_4_ groups, providing a spacious open structure and highly ionized anions, which are beneficial for the transport and diffusion of sodium ions. Additionally, these materials have a high porosity rate, which can offer more storage space for sodium ions. They exhibit a high reversible capacity and long life cycle and have been studied for many years in SIBs [82,83,84]. In NASICON-type SIB cathodes, in-depth studies have been conducted on Na_3_V_2_(PO_4_)_3_ (NVP) and Na_3_V_2_O_2_x(PO_4_)_2_F_3–2_x. Expanding the cell unit of NVP is one effective method to achieve rapid sodium ion transport. In the literature, larger ion doping is often used to expand the volume of the cell unit. For instance, a small amount of Nb ions (Nb^5+^) were doped into NVP without altering its structure, thus expanding the volume of NVP [85]. Meanwhile, the optimized Na_3_V_1.9_Nb_0.1_(PO_4_)_3_/C material exhibits high specific capacity (~114 mAh g^−1^ at 0.5 C), superior rate capability (~100 mAh g^−1^ at 20 C), and excellent long-term cycling stability (the specific capacity after 1000 cycles at 50 °C is 74.7 mAh g^−1^, corresponding to a capacity retention rate of 0.0258% per cycle).

**Figure 6:**
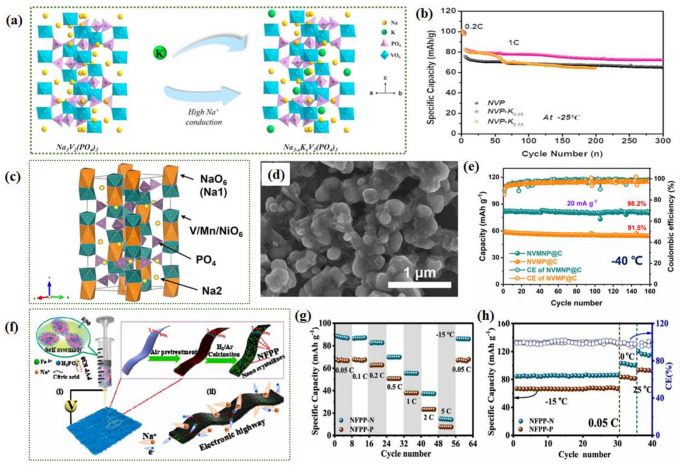
(**a**) A schematic illustration of the structural stabilization of NVP electrode materials by doping K ions into the Na sites. (**b**) Cycle performance at 1 C of NVP, NVP-K_0.05_, and NVP-K_0.10_ samples under the low temperature of −25 °C. Reproduced with permission from ref. [87]. Copyright 2023 Elsevier Ltd. (**c**) Crystal structure of the as-constructed Na_4_VMn_0.7_Ni_0.3_(PO_4_)_3_. (**d**) SEM of the NVMNP@C material. (**e**) Cycle performance (−40 °C) of NVMNP@C and NVMP@C cathodes at 20 mA g^−1^. Reproduced with permission from ref. [88]. Copyright 2024 The Authors. (**f**) Schematic illustration (fi) of the synthesis of NFPP–N and (fii) the electronic and Na^+^ diffusion paths on one-dimensional NFPP nanoribbons. (**g**) Rate performances of NFPP–N and NFPP–P at −15 °C. (**h**) The cycling performances of NFPP–N and NFPP–P at 0.05 C at −15, 0, and 25 °C. Reproduced with permission from ref. [89]. Copyright 2021 Elsevier B.V.

**Figure 7:**
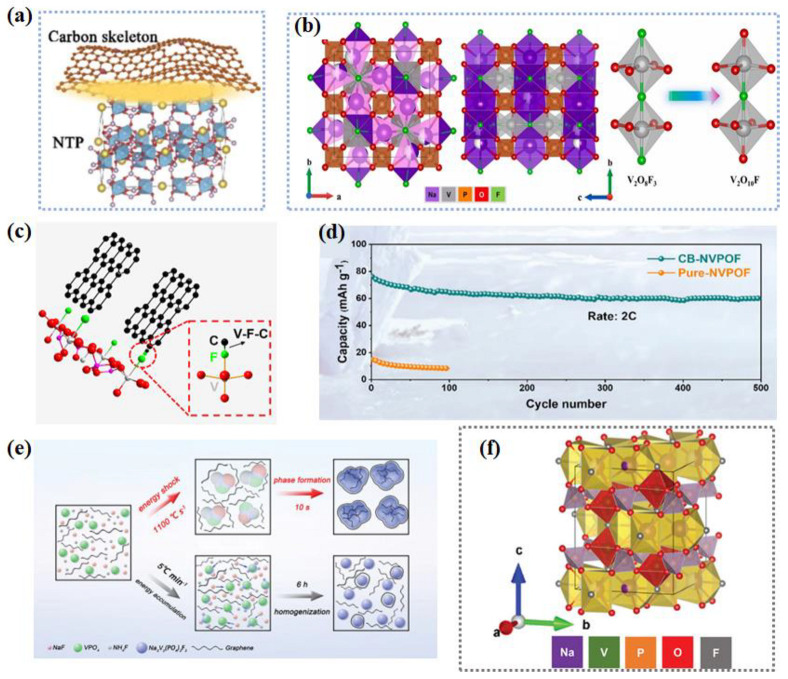
(**a**) A schematic illustration of the electronic coupling between NTP and in situ generated carbonaceous skeleton. Reproduced with permission from ref. [90]. Copyright 2022 Elsevier Inc. (**b**) Schematic illustration of the crystal structure of Na_3_V_2_(PO_4_)_2_F_3_. Reproduced with permission from ref. [93]. Copyright 2020 Elsevier Ltd. (**c**) Diagram of the V−F−C bonding between NVPOF and graphene. (**d**) Cycling performance at 2 C at −40 °C of the pure NVPOF and CB-NVPOF at −40 °C. Reproduced with permission from ref. [94]. Copyright 2023, American Chemical Society. (**e**) Schematic illustration of the formation mechanism of the NVPF formed by HTS and tube furnace (TF). (**f**) A schematic illustration of the crystal structure of NVPF. Reproduced with permission from ref. [95]. Copyright 2024 Wiley-VCH GmbH.

Regarding LT performance, in the early stages, experiments were conducted replacing Na^+^ with K^+^ and demonstrated an increase in the battery volume [86]. More recently, to address the issue of poor rate performance of the Na_3_V_2_(PO_4_)_3_ electrode at LTs due to structural damage during the insertion/extraction of Na ions, an appropriate amount of K ions were introduced into Na_3_V_2_(PO_4_)_3_ through a rational design [87]. This increased the unit cell volume, widened the Na ion migration channels (Figure 6a), and ensured that the Na_3_V_2_(PO_4_)_3_ electrode exhibits good electrochemical performance over a wide temperature range (−25 °C to 40 °C). Furthermore, Na_2.95_K_0.05_V_2_(PO_4_)_3_ maintains a capacity of 72 mAh g^−1^ even at −25 °C, while Na_3_V_2_(PO_4_)_3_ without K doping has a lower capacity (Figure 6b). Additionally, due to the high cost of vanadium raw materials in NASICON, there is a growing emphasis on finding alternative active transition metals to achieve similar effects while reducing cost pressures. Xu et al. employed bimetallic (Mn and Ni) substitution to improve its conductivity and designed a novel low-vanadium Na_4_VMn_0.7_Ni_0.3_(PO_4_)_3_@C (NVMNP@C) SIB cathode [88]. Figure 6c shows the crystal structure model of Na_4_VMn_0.7_Ni_0.3_(PO_4_)_3_. In this 3D open framework, the interconnected Na^+^ channels are constructed by an insular Ni/Mn/VO_6_ octahedral sharing all the corners with a PO_4_ tetrahedra. In Figure 6d, the overall NVMNP@C particles exhibit an ellipsoidal shape with dimensions ranging from 100 to 300 nm, and the surficial-coated carbon layer, which is derived from the carbonization of sucrose and citric acid, can be clearly observed. This cathode demonstrates outstanding LT adaptability (−40 °C); after 230 cycles at 100 mA g^−1^, it retains 90.4% of its capacity (Figure 6e). The utilization of highly conductive matrices, such as one-dimensional carbon nanoribbons [89] and tablet-like carbon [90], to construct fine structures within NASICONs to enhance their electrochemical performance is also commonly mentioned in the literature. Zhang et al. employed polyvinylpyrrolidone-assisted electrospinning to prepare nano Na_4_Fe_3_(PO_4_)_2_P_2_O_7_ nested on carbon nanoribbons [89] (Figure 6f), improving its electronic conductivity and LT performance. Figure 6g demonstrates the rate capabilities of NFPP–N and NFPP–P electrodes at LTs (−15 °C), It is worth noting that at −15 °C, the nanoribbon of Na_4_Fe_3_(PO_4_)_2_P_2_O_7_ can provide a discharge capacity of 84.5 mAh g^−1^ at 0.05 C (Figure 6h). In another study, sodium titanate phosphate (NaTi_2_(PO_4_)_3_, NTP) nanocubes were in situ decorated onto sheet-like carbon (NTP/C) [90]. The interaction between NTP and the decorated carbonaceous skeleton is depicted in Figure 7a, exhibiting excellent LT sodium storage performance. After 200 cycles at −25 °C under 0.5 A g^−1^, it still maintained a stable capacity (94.3 mAh g^−1^). Additionally, introducing Ni^2+^ [91] or Mn^2+^ [92] into NaFePO_4_ (NFP) to enhance its electrochemical performance is another measure that can inspire improvements in the LT performance of SIBs.

Na_3_V_2_(PO_4_)_2_F_3_ (NVPF) is a promising class of polyanionic materials, with a theoretical operating voltage of 3.85 V and a theoretical specific capacity of 128.3 mAh g^−1^ [93]. The high theoretical energy density of 507 Wh kg^−1^ is comparable to that of LiFePO_4_ (≈ 530 Wh kg^−1^), which illustrates the practical application prospect of NVPF. The three-dimensional NVPF framework provides more Na^+^ transport paths and improves the stability of the structure. However, it still has the common problem of phosphate materials, and the electronic conductivity is low. Strategies such as nanoscale particles [96], morphological design (e.g., hollow microspheres, nanoflowers, etc.) [97,98], and doping [99] have been used to improve their electronic conductivity and ion mobility. The work of Deng et al. discussed the reasons for the formation of a 3.3 V low-voltage platform during NVPF operation, and they proposed that the formation of the low-voltage platform was caused by the additional formation of [VO_6_] caused by fluorine loss during the synthesis process [93]. They also proposed a strategy to stabilize fluorine, which effectively improved the structural stability and kinetic properties of the material (Figure 7b) and had good LT performance (−25~55 °C). Xu et al. solved the interface problem of NVPF by establishing chemical bonds [94] and developed an interface V−F−C bonding NVPOF (CB-NVPOF) (Figure 7c). The developed CB-NVPOF has excellent LT performance, maintaining about 80% capacity after 500 cycles at 2 C (Figure 7d). Soon after, a large conductive NVPF for a high tap density cathode was synthesized using a high-temperature shock (HTS) strategy (Figure 7e) [95]. The synthetic material has a wide temperature adaptability of −45~55 °C, further proving the practical application prospect of HTS-NVPF. A lot of full-cell research has been conducted. A superior LT SIB composed of 3D porous Na_3_V_2_(PO_4_)_3_/C (NVP/C-F) and NaTi_2_(PO_4_)_3_/C foams (NTP/C-F) has been developed (Figure 7f) [100]. The designed NTP/C-F||NVP/C-F full cell exhibits excellent LT kinetics (−20 °C) and cycle stability. In summary, heteroatom doping, bimetallic substitution, introducing a carbon framework, establishing chemical bonds, and innovative synthetic methods are widely used to enhance the LT performance of polyanionic compounds in SIBs. Future promising directions include optimizing the structure of polyanionic compounds to enhance conductivity, exploring new synthesis methods for better performance, and investigating hybrid materials that combine polyanionic compounds with other active materials to further boost battery efficiency and lifespan.

#### 2.2.2. Prussian Blue and Prussian Blue Analogs

**Figure 8:**
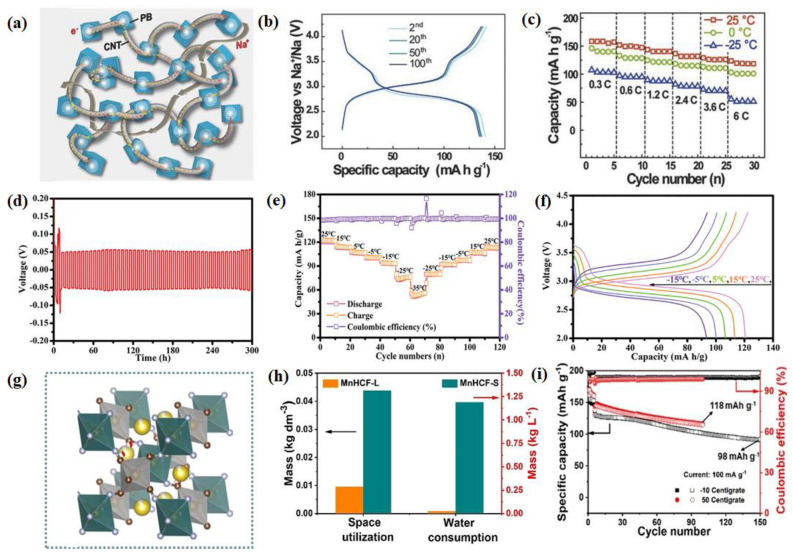
(**a**) Schematic illustration showing the mixed conducting network in the PB/CNT. (**b**) GDC profiles at 0.1 C of the PB/CNT cathode at −25 °C for 100 cycles. (**c**) Rate capabilities of the PB/CNT cathode at different temperatures. Reproduced with permission from ref. [112]. Copyright 2016 WILEY-VCH Verlag GmbH & Co. KGaA, Weinheim. (**d**) Galvanostatic cycling test of Na symmetric cells at a current density of 0.5 mA cm^−2^. (**e**) Low-temperature cycling test of SSIBs. (**f**) Corresponding charge–discharge curves of SSIBs. Reproduced with permission from ref. [113]. Copyright 2019 WILEY-VCH Verlag GmbH & Co. KGaA, Weinheim. (**g**) Schematic illustrations of the structures of the monoclinic MnHCF-S-170. (**h**) Space utilization and water consumption comparison of the as-prepared samples. (**i**) Long-term cycling performances at −10 °C and 50 °C at the current density of 100 mA g^−1^. Reproduced with permission from ref. [114]. Copyright 2022 Wiley-VCH GmbH.

As a simple cyanide-coordinated polymer, Prussian blue (PB) and Prussian blue analogs (PBAs) are considered promising positive electrode materials for SIBs due to their three-dimensional open frameworks, adjustable structures, and chemical compositions. However, due to structural defects, crystalline water, and interface instability, current half-cell experiments have difficulty in simultaneously providing a high capacity, high rate capability, and long life cycle for both PB and PBAs [101,102]. Due to the instability of bulk and interface structures, PB and PBAs sometimes sacrifice life cycle to achieve high capacity [103,104]. In recent years, significant progress has been made in optimizing the bulk and interface structures of PB and PBAs through various improvement strategies [105,106,107,108]. PB has been proven to exhibit small volume changes, high energy density, and excellent rate capability when cycled in ambient-temperature sodium half-cells [109,110,111]. Earlier, You et al., used low-cost positive electrode material PB with excellent Na storage properties to form solid composite materials (PB/CNT) by nucleating cubic nanoparticles on carbon nanotubes (CNTs) [112]. The uniform PB nanocrystals threaded by a CNT intertwined mat retain good electric contact between the PB particles and the current collector for lowering the temperature (Figure 8a). Subsequent experiments have demonstrated its rapid and stable electrochemical cycling at a wide temperature range. As shown in Figure 8b, the PB/CNT can still deliver a capacity of 142 mA h g^−1^ and a specific energy of 408 W h kg^−1^ at −25 °C (0.1 C), as well as a negligible capacity loss and increase in voltage polarization after 100 cycles. At 2.4 C, the PB/CNT cathode still retains reversible capacities of 114 and 88.4 mAh g^−1^ at 0 and −25 °C, respectively, which are 87% and 67% of the capacities delivered at 25 °C. Even at a high rate of 6 C, an 83% capacity retention was observed at 0 °C compared with 25 °C (Figure 8c).

To increase the electric performance of the PB cathode, a solid-state polymer electrolyte (PFSA-Na membrane) for solid-state sodium batteries (SSIBs) has been investigated. As shown in Figure 8d, the stable galvanostatic cycling performance for 300 h further illustrates that the PFSA-Na membranes display both excellent mechanical and electrochemical stability. Furthermore, they exhibit an excellent LT performance of −35 °C (Figure 8e,f), and this provides additional evidence of the excellent LT electrochemical properties of the PFSA-Na membranes [113]. Peng et al. [114] designed a “water-in-salt” nanoreactor to prepare highly crystallized Mn-based PBAs (Figure 8g). More importantly, the water consumption of the as-designed “water-in-salt” nanoreactor was nearly 50 times higher than that of the typical co-precipitation method in water (from 0.024 kg L^−1^ to 1.188 kg L^−1^), indicating that this nanoreactor method could be applied for low-cost and high-efficiency synthesis of PBAs in large-scale practical production (Figure 8h). The as-prepared Mn-based PBA (MnHCF-S-170) shows fast and stable electrochemical performance over the wide temperature range from −10 °C to 50 °C and enhanced air stability [114]. In more detail, it shows the discharge capacity of 98 mAh g^−1^ after 150 cycles at −10 °C (Figure 8i). In summary, recent advancements have demonstrated promising enhancements in electrochemical performance and synthesis efficiency for positive electrode materials in SIBs. Innovative approaches include solid composite PB/CNT materials for improved LT stability and “water-in-salt” nanoreactors for scalable production of Mn-based PBAs with superior electrochemical properties across a wide temperature range.

#### 2.2.3. Layered Transition Metal Oxides

**Figure 9:**
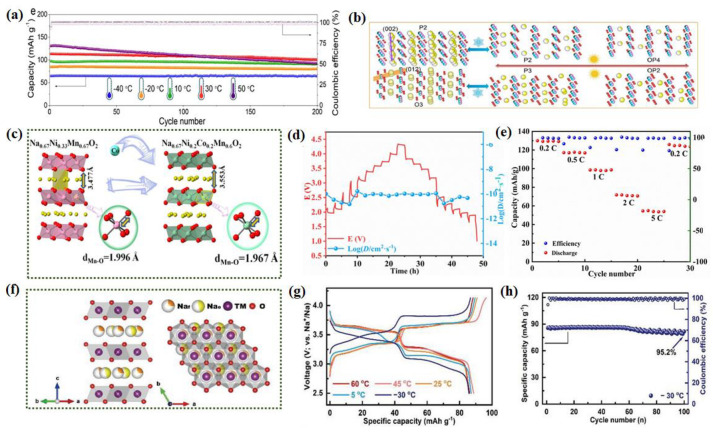
(**a**) Cycling performance of P_2_/O_3_-NaMnNiCuFeTiOF at different temperatures. (**b**) Schematic diagram of structural evolution of P_2_/O_3_-NaMnNiCuFeTiOF. Reproduced with permission from ref. [117]. Copyright 2023 Elsevier B.V. (**c**) Schematic illustration of effect of Co^3+^ doping on electrode structure. (**d**) GITT curves and the corresponding D_Na_^+^ values for the NNCM cathode material at −40 °C. (**e**) Rate capabilities at different rates from 0.2 to 5 C at −40 °C. Reproduced with permission from ref. [118]. Copyright 2021 Elsevier B.V. (**f**) P_2_-type crystal structure viewed along a axis (left) and c axis (right). (**g**) Typical charge/discharge profiles of NM-2 at 60, 45, 25, 5, and −30 °C. (**h**) Cycle performance at −30 °C of NM-2. Reproduced with permission from ref. [119]. Copyright 2023 Wiley-VCH GmbH.

Due to the simplicity of preparation, layered transition metal oxides (Na_x_TMO_2_, where 0 < x < 1, TM represents 3D transition metals) are considered highly promising cathode materials. On the other hand, these materials also face challenges such as complex phase transitions during charge and discharge, transition metal migration and dissolution, and instability in air. Layered NaxMn_1-y_M_y_O_2_ oxides based on Mn (where M = Ni, Co, Fe, Ti, Zn, Li, Mg, Cu, etc.) and their derivatives have garnered special attention due to their high theoretical capacity, simple preparation, and eco-friendliness [115,116]. Researchers have synthesized a high-entropy biphasic Na_0.7_Mn_0.4_Ni_0.3_Cu_0.1_Fe_0.1_Ti_0.1_O_1.95_F_0.1_ cathode material [117]. After 200 cycles, P_2_/O_3_-NaMnNiCuFeTiOF exhibits a capacity retention of 99.5, 94.5, 93.3, 88.9, and 71.1% at −40, −20, 10, 30, and 50 °C, respectively (Figure 9a), suggesting impressive cyclic stability in a wide temperature range. The excellent Na-storage performance of P_2_/O_3_-NaMnNiCuFeTiOF is attributed to the designed high entropy and biphasic structure, which can improve the structural stability by relieving the Jahn–Teller distortion and avoiding the sliding of TM layers in the wide temperature range (Figure 9b). Another study employed a cosubstitution strategy, introducing Co into Na_0.67_Ni_0.33_Mn_0.67_O_2_, effectively enlarging the interlayer spacing of TMO_2_ and accelerating Na^+^ diffusion kinetics [118] (Figure 9c). The optimized Na_0.67_Ni_0.2_Co_0.2_Mn_0.6_O_2_ (NNCM) exhibited good long-term cycling stability at LTs. The specific diffusion coefficient value of NNCM is shown in Figure 9d. The D_Na_^+^ values of NNCM only drop slightly at LTs with a range from 10^−11^ to 10^−10^ cm^2^ s^−1^. Interestingly, the fluctuation range of D_Na_^+^ value at LTs is smaller than that at room temperature, indicating that the transport rate of sodium ions in the whole charge/discharge processes is more stable at LTs. Similarly, galvanostatic charge/discharge (GCD) cycling tests were also performed under LT. P_2_-NNCM shows reversible discharge capacities of 132.2, 117.5, 98.2, 70.5, and 54 mAh g^−1^ at 0.2, 0.5, 1, 2, and 5 C (Figure 9e). Moreover, the doping of other elements can also inhibit phase transition and enhance conductivity by accommodating more Na ions at the e-site (Na_e_) to regulate the ratio of Na_e_/Na_f_ in P_2_-type Na_2/3_Ni_1/3_Mn_2/3_O_2_ (Figure 9f) to obtain the Na_0.696_Ni_0.329_Mn_0.671_O_2_ (NM-2) [119]. The highest Na_e_/Na_f_ value (1.6914) shows the most excellent capacity retention (95.2−65.0%) in a wide temperature range (−30 to 60 °C) (Figure 9g). With a lower diffusion energy barrier (0.8301 eV for edge sites and 1.0664 eV for face sites), the Na-ion has faster ion migration in edge sites than face sites. As a result, the P_2_-type NNMO remains about 95.2% capacity after 100 cycles at 1 C (170 mA g^−1^) at −30 °C without any activation charging process at RT (Figure 9h). In conclusion, layered transition metal oxides exhibit robust performance enhancements at LT through the above strategies. High-entropy biphasic Na_0.7_Mn_0.4_Ni_0.3_Cu_0.1_Fe_0.1_Ti_0.1_O_1.95_F_0.1_ and cosubstituted Na_0.67_Ni_0.2_Co_0.2_Mn_0.6_O_2_ cathodes demonstrate impressive cyclic stability across a wide range from −40 °C to 50 °C, attributed to structural stability improvements and enhanced Na^+^ diffusion kinetics. Doping strategies further enhance conductivity and inhibit phase transitions, exemplified by Na_0.696_Ni_0.329_Mn_0.671_O_2_, achieving superior capacity retention from −30 °C to 60 °C. These advancements underscore the potential of LT-tailored designs in advancing SIB technologies for diverse climate applications. But at present, the number of studies in this direction needs to be improved, and it is a promising research direction that researchers need to explore.

### 2.3. Electrolyte

The electrolyte is a crucial component of SIBs, primarily composed of solvents, electrolyte salts, and additives. During the charge and discharge processes of the battery, the electrolyte acts as an ion conductor, facilitating the transfer of Na^+^ ions between the positive and negative electrodes, and serving as a bridge connecting them. Additionally, the electrolyte directly participates in forming the SEI film on the electrode surfaces. Improving the LT performance of the battery through modified electrolytes is currently one of the most economical and effective approaches.

Compared to traditional LIBs, the electrolyte of SIBs can maintain higher ion conductivity at LTs, possibly due to the higher concentration of salts in the electrolyte. Researchers have conducted extensive studies on LT electrolytes and found that electrolytes can effectively enhance LT performance. The main issue with SIBs at LTs is the slow diffusion process, including the diffusion of Na^+^ at the SEI and the charge transfer process of Na^+^ and e^−^ at the electrode/electrolyte interface. These processes are hindered by temperature reduction, leading to a significant decrease in the battery’s kinetic performance and severely affecting the electrochemical performance of SIBs [120]. Therefore, research on LT SIBs cannot be separated from the study of electrolytes. Currently, non-aqueous liquid electrolytes offer a vast solvent pool that can be adapted to different active materials and electrochemical evolution windows, making them more suitable for use as LT electrolytes. The following will discuss the research progress of electrolytes for LT SIBs, especially non-aqueous liquid electrolytes.

#### 2.3.1. Ester-Based Electrolytes

Currently, common solvents in SIB electrolytes are esters and ethers. However, ester-based electrolytes (such as ethylene carbonate, EC; dimethyl carbonate; diethyl carbonate; etc.) are becoming increasingly popular because traditional ether-based electrolytes are unstable at voltages above 4 V, posing challenges in matching high-voltage cathodes [121]. Carbonate-based electrolytes are indeed suitable choices for SIBs due to their high salt solubility, rapid ion transport properties, and strong electrochemical stability [122]. In recent years, many researchers have been attempting to improve the LT performance of carbonate-based electrolytes. Zheng et al. employed a cosolvent strategy to make Na||Na_3_V_2_(PO_4_)_2_O_2_F (Na||NVPOF) batteries resilient to LTs down to −30 °C [11]. Che et al. focus on engineering aspects to optimize electrolytes. The basic physicochemical properties were systematically measured, including the ionic conductivity, viscosity, wettability, and thermochemical stability of the electrolytes, using NaPF_6_ as the solute and the mixed solvent with different components of EMC, DMC, or DEC in PC or EC [123]. Additionally, Deng et al. proposed the spontaneous formation of weakly solvated structures in low-concentration electrolytes [124].

**Figure 10:**
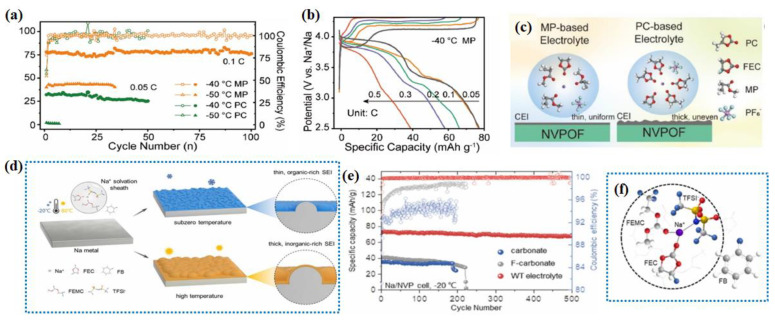
(**a**) Long-term cycling performance for NVPOF cathodes at −40 and −50 °C. (**b**) Rate capability for NVPOF cathode at −40 °C. (**c**) Schematic illustration of the formed CEI in different electrolytes. Reproduced with permission from ref. [127]. Copyright 2024 Published by Elsevier Ltd. on behalf of the editorial office of the *Journal of Materials Science & Technology*. (**d**) Schematic illustration of Na growth behavior and temperature-responsive SEI in the WT electrolyte at subzero and high temperature. (**e**) Representative solvation sheath determined by MD simulations. The Na, C, O, H, F, S, and N are marked with purple, gray, red, white, light blue, yellow, and navy blue, respectively. (**f**) Long-term cycling performance of Na/NVP cells operated in the corresponding electrolytes at 0.5 C, −20 °C. Reproduced with permission from ref. [128]. Copyright 2022 Elsevier Ltd.

However, due to the high melting point and viscosity of carbonates, it is challenging to reach lower operating temperatures. Therefore, the development of an electrolyte capable of withstanding extreme LTs remains crucial and urgent. There are some reports of using fluorinated carboxylic esters in LIBs’ electrolytes for LT purposes [125,126]. The anodic stability might be enhanced compared with carboxylic acid without the fluoride substitution. Their use might inspire the electrolyte field for SIBs. Liu et al. have developed a polyethylene glycol-based SIB electrolyte, incorporating fluorinated ethylene carbonate (FEC) as a film-forming agent [127]. This electrolyte possesses a low melting point, sufficient ion conductivity, and high electrochemical stability at LTs. The resulting Na||Na_3_V_2_(PO_4_)_2_O_2_F cell demonstrates a capacity retention of 96% at −40 °C (after 100 cycles at 0.1 C) (Figure 10a) and a superior rate performance at LTs (Figure 10b). Furthermore, it has been confirmed that the carboxylate-based electrolyte promotes the formation of a robust and uniform cathode/electrolyte interface layer (Figure 10c), accelerating ion diffusion kinetics and thus collectively contributing to excellent LT performance. The selection of fluorinated solvents is also an effective strategy to enhance the LT performance, which makes it easy to form a fluorinated inorganic-rich SEI on the surface of the anode, which possesses a higher ionic conductivity. Liu et al. used a 1.0 M NaTFSI-FEC/FEMC/fluorobenzene (FB) (3:3:4 by volume) as an LT electrolyte that combines low viscosity and high ionic conductivity [128]. FB can weaken the coordination of the Na^+^ solvent (Figure 10d), thereby mitigating the decomposition of solvent molecules on the surface of the SMBs to form a thin and inorganic-enriched SEI, which also allows the Na_3_V_2_(PO_4_)_3_ anode to exhibit a much higher specific capacity (73.9 mAh g^−1^) and longer life cycle (−20 °C for 500 cycles) than the EC electrolyte (Figure 10e). As shown in Figure 10f, the primary Na^+^ coordination shell was taken up by the FEC, FEMC, and TFSI^−^, revealing that the FB molecules hardly interact with Na^+^ and do not enter the first solvation sheath due to its poor solvating ability. High fluorine content electrolytes using FEC as a high dielectric solvent component facilitate the LT performance of SIBs.

**Figure 11:**
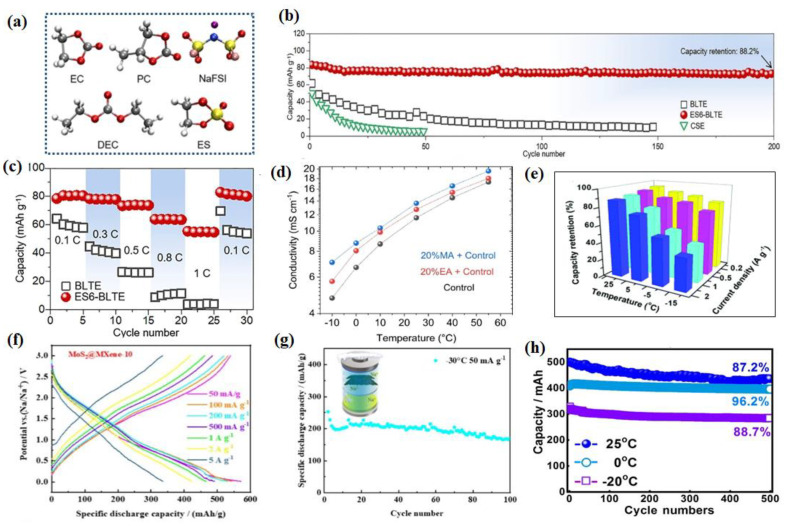
(**a**) The designed new low-temperature electrolyte system of ES_6_-BLTE. (**b**) Cyclability of the Na||NVP cells with CSE, BLTE, and ES_6_-BLTE electrolytes at 0.1 C at −40 °C. (**c**) Rate performance of Na||NVP cells at −40 °C. Reproduced with permission from ref. [129]. Copyright 2023 Wiley-VCH GmbH. (**d**) Ionic conductivity of the electrolyte blends: control (1 M NaPF_6_ in EC-PC-DMC 1:1:2 by vol%), 20%MA + control (1 M NaPF_6_ in EC-PC-DMC-MA 1:1:2:1 by vol%), and 20%EA + control (1 M NaPF_6_ in EC-PC-DMC-EA 1:1:2:1 by vol%) measured at different temperatures. Reproduced with permission from ref. [130]. Copyright 2022 Elsevier Ltd. (**e**) The capacity retentions of Sb_2_Se_3_/rGO along with temperature variations between 25 and −15 °C at various current densities of 0.2, 0.5, 1, and 2 A g−1. Reproduced with permission from ref. [57]. Copyright 2022 Royal Society of Chemistry. (**f**) Charge–discharge voltage profiles at various current densities of MoS_2_@MXene@D-TiO_2_ electrodes. (**g**) Low-temperature cycling performance of MoS_2_@MXene@D-TiO_2_ electrodes. Reproduced with permission from ref. [59]. Copyright 2022 American Chemical Society. (**h**) The cycling stability during 500 cycles at different operation temperatures of the 500 mAh soft-packed Na_3_V_2_(PO_4_)_3_//Na_3_V_2_(PO_4_)_3_ symmetrical battery. Reproduced with permission from ref. [131]. Copyright 2021 The Chemical Industry and Engineering Society of China, and Chemical Industry Press Co., Ltd.

The utilization of additives is also a common strategy. For instance, Zhong et al. introduced ethyl sulfate (ES) additives into 1.0 M NaFSI-ethylene carbonate (EC)/propylene carbonate (PC)/diethyl carbonate (DEC) (denoted as BLTE) to regulate the solvation structure [129], and the new LT electrolyte system of ES (6 vol%) + BLTE is developed (denoted as ES_6_-BLTE) (Figure 11a). Figure 11b shows the cyclability of different batteries at 0.1 C at −40 °C, for the ES_6_-BLTE electrolyte, and only slight capacity degradation is observed, displaying a high-capacity retention of 88.2% after 200 cycles. The rate performance measurement (Figure 11c) shows that the Na||NVP battery with ES_6_-BLTE can operate at a high rate of 1 C with a high-capacity retention of 55 mAh g^−1^, while that of BLTE almost fails at 0.8 C. In another study, researchers explored the impact of adding methyl acetate (MA) and/or ethyl acetate (EA) on the Na-ion electrolyte’s conductivity and electrochemical performance of our reference electrolyte containing four additives, namely sodium oxalato(difluoro)borate (NaODFB), vinylene carbonate (VC), succinonitrile (SN), and tris-trimethylsilylphosphite (TMSPi) [130]. As shown in Figure 11d, the bulk ionic conductivity of the control electrolyte was measured as a function of temperature. And their LT (0 °C) performance was also discussed. However, this co-solvent is still not perfect as it induces some lifetime penalties at high temperatures as compared to a co-solvent free electrolyte containing additives.

In electrochemical testing, ester-based electrolytes are often used to ensure the good LT performance of electrode materials. Hu et al. synthesized a Sb_2_Se_3_/rGO anode, using 1 mol L^−1^ NaClO_4_ in EC:DMC at room temperature [57], and this anode delivered the capacities of 580.6, 547, 517.5, 498.8, 467.9, and 396.8 mAh g^−1^ at 100, 200, 500, 1000, and 2000 mA g^−1^, respectively. However, at −15 °C, the capacity retention was 77.5% at 200 mA g^−1^ (Figure 11e). Zhang et al. developed a high-performance anode material based on MoS_2_, Ti_3_C_2_T_x_ MXene and dual-phased-TiO_2_ (MoS_2_@MXene@D-TiO_2_), which has a high discharge capacity and rate capability [59]. Figure 11f illustrates the voltage profile of MoS_2_@MXene@D-TiO_2_||Na with 1 mol L^−1^ NaClO_4_ in EC:DEC (1:1 vol%) at different current densities. At room temperature, the material delivered an outstanding capacity of 571.6, 545.5, 497.3, 466.5, and 359.6 mAh g^−1^ at 50, 100, 500, 1000, and 5000 mA g^−1^, respectively. However, its performance significantly decreased when the cell was taken to −30 °C, as shown in Figure 11g. At −30 °C, 50 mA g^−1^, MoS_2_@MXene@D-TiO_2_ could only deliver a capacity of 253 mAh g^−1^, which was only 44.2% of the RTC. Cui et al. used a 0.8 mol L^−1^ NaPF_6_/EMC + PC + FEC (50:48:2, *v*/*v*/*v*) electrolyte to test the electrochemical performance of a porous Na_3_V_2_(PO_4_)_3_/C cathode material [131]. As shown in Figure 11h, the NNB displayed an outstanding cycling performance at different temperatures. It is worth noting that the Na_3_V_2_(PO_4_)_3_//Na_3_V_2_(PO_4_)_3_ symmetrical battery has a capacity retention of 88.7% after 500 cycles at −20 °C. Highly dispersed maricite NFP nanoclusters (NFPNCs) with ultrafine NFP@C subunits (3 nm) were designed and synthesized by Liu et al., and the electrolyte was 1 M NaClO_4_ dissolved in anhydrous PC with 5 vol% FEC. Even at LT (−10 and −20 °C), the NFPNCs still exhibited 85.5% and 75.8% capacity retention at −10 and −20 °C, respectively [132]. All in all, this section focuses on modifying ester-based electrolytes, employing fluorides to enhance electrode stability and additives to adjust electrolyte composition for broader temperature adaptability. Ester-based electrolytes are widely utilized across diverse electrochemical systems, including half-cell and full-cell tests, where they exhibit remarkable performance in LT environments.

#### 2.3.2. Ether-Based Electrolyte

**Figure 12:**
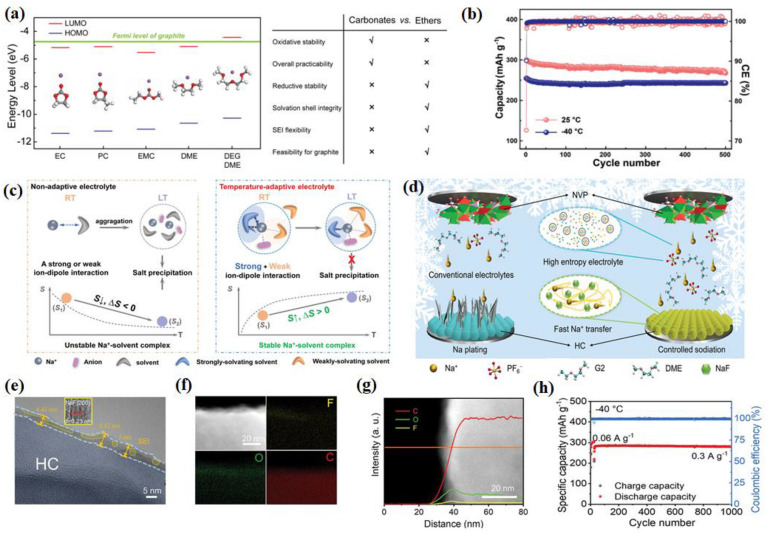
(**a**) LUMO and the highest occupied molecular orbital (HOMO) level of Na^+^-EC, Na^+^-PC, Na^+^-EMC, Na^+^-DME, and Na^+^-DEGDME complexes. Reproduced with permission from ref. [133]. Copyright 2021 Wiley-VCH GmbH. (**b**) Cycling performance of the cells equipped with NDT electrolyte at 25 and −40 °C, at a current density of 100 mA g^−1^. (**c**) Schematic illustration showing the low-temperature electrolyte design strategy. The solvation structure variations with temperature are driven by entropy change: non-adaptive electrolyte (left) and temperature-adaptive electrolyte (right). Reproduced with permission from ref. [135]. Copyright 2023 Wiley-VCH GmbH. (**d**) Schematic illustration of SIBs with conventional electrolytes (left) and the designed electrolyte (right) operating at low temperatures. Characterizations of the SEI film for the cycled HC anode with 1 m NaPF_6_-G_2_/DME electrolyte at −40 °C. (**e**) HRTEM image of the SEI film on HC anode. STEM images of (**f**) C, O, F elements mapping and (**g**) line scan of the SEI film. (**h**) Cycling stability of HC||Na half-cell with 1 m NaPF_6_-G_2_/DME electrolyte at 0.30 A g^−1^, −40 °C. Reproduced with permission from ref. [136]. Copyright 2024 Wiley-VCH GmbH.

**Figure 13:**
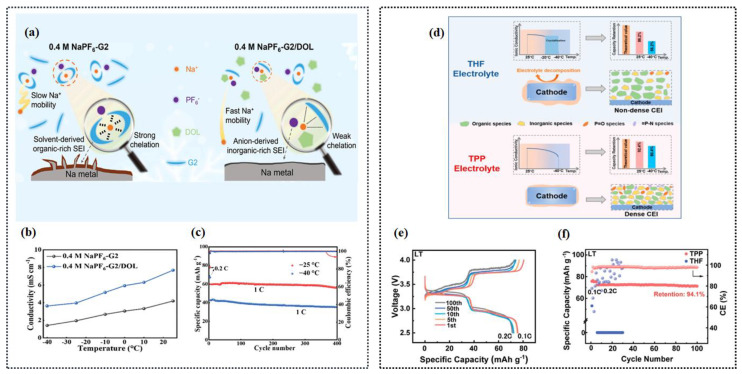
(**a**) Schematic illustration of the mechanism of improved LT Na reversibility by the DOL-diluted electrolyte. (**b**) Ionic conductivity of 0.4 m NaPF_6_-G_2_ and 0.4 m NaPF_6_-G_2_/DOL electrolytes. (**c**) Long-term galvanostatic cycling performance of AFSMBs at 1 C under −25 °C and −40 °C. Reproduced with permission from ref. [137]. Copyright 2024 Wiley-VCH GmbH. (**d**) A schematic illustration of the working mechanism of the selected electrolyte in the MN cathode. (**e**) GCD curves of MN cathode with TPP electrolyte for different cycles at −40 °C. (**f**) Cycling performance of MN cathode in TPP and THF electrolytes at −40 °C. Reproduced with permission from ref. [138]. Copyright 2023 American Chemical Society.

Regarding ether-based electrolytes, it is important to note that the solvation structure must possess a strong anti-reduction ability to enable reversible co-intercalation without causing electrode exfoliation due to electrolyte decomposition. Overall, the physicochemical properties of ether-based electrolytes make them suitable for LT applications and their enhanced kinetic performance further contributes to their outstanding LT performance. From the aspect of interphase properties, compared with carbonates, sodium–ether complexes have higher cathodic stability. According to the calculation results of Liang et al., Na^+^-DEGDME complexes have higher lowest unoccupied molecular orbital (LUMO) levels than the Fermi level of graphite and Na^+^-carbonates [133], which indicates the high anti-reduction ability of ether-based electrolytes (Figure 12a). Inorganic-rich, thinner, and ionic conductive interphase layers and a lower RSEI are more commonly observed with ether-based electrolytes than carbonate-based electrolytes at room temperature [134]. Yang and his co-workers discussed NaPF_6_ dissolved in a mixture of strongly solvated (SS) and weakly solvated (WS) ether solvents [135], and it was demonstrated that the designed electrolytes could achieve a stable SEI and high Na^+^ mobility at the electrode/electrolyte interface. As shown in Figure 12b, the capacity retention at 25 °C after 500 cycles is 89.1%, and the capacity retention at −40 °C after 500 cycles is 95.4%. Interestingly, the cycling stability of HC||Na cell at LT (−40 °C) seems to be even better than that at room temperature, making our electrolyte a decent candidate for Na-ion battery operating in a wide temperature range from −50 to 60 °C with the temperature-adaptive feature. The solvation structure in these electrolytes can be spontaneously transformed at LT to avoid salt precipitation, giving the electrolytes a temperature-adaptive property (Figure 12c). This property guarantees a significant improvement in the performance of SIBs at LTs, improving the performance of the batteries. A LT, useful, high-power-density rechargeable Na_3_V_2_(PO_4_)_3_ || hard carbon (HC) sodium-ion full battery without Na plating is realized by electrolyte regulation by Wang et al. (Figure 12d). As shown in Figure 12e–g, the average thickness of the SEI layer formed on the hard carbon (HC) surface is only 5.08 nm, which is consistent with the low RSEI value and hence accounts for the fast interfacial Na^+^ transport kinetics at LTs [136]. At the same time, F and O elements are abundantly distributed on the surface of HC. At −40 °C, after 1000 cycles, HC retained a specific capacity up to 280.70 mAh g^−1^ with a capacity retention of 99.50% (Figure 12h), which shows that the designed electrolyte has an excellent LT performance. The solvation structure of the conventional 1 m NaPF_6_ in diglyme (G_2_) electrolyte by facile cyclic ether (1,3-dioxolane, DOL) dilution is efficiently reconfigured for high Na reversibility at LTs (Figure 13a). DOL molecules alleviate the Na^+^-PF_6_^−^Coulombic interaction and intermolecular forces of G_2_ solvents, generating a remarkably high Na^+^ mobility [137] (Figure 13b). As depicted in Figure 13c, an initial discharge capacity of 59.7 mAh g^−1^ and capacity retention of 93.3% over 400 cycles at −25 °C were achieved. A capacity retention of 82.5% over 400 cycles at −40 °C and 91% over ≈140 cycles at −55 °C were also realized.

**Figure 14:**
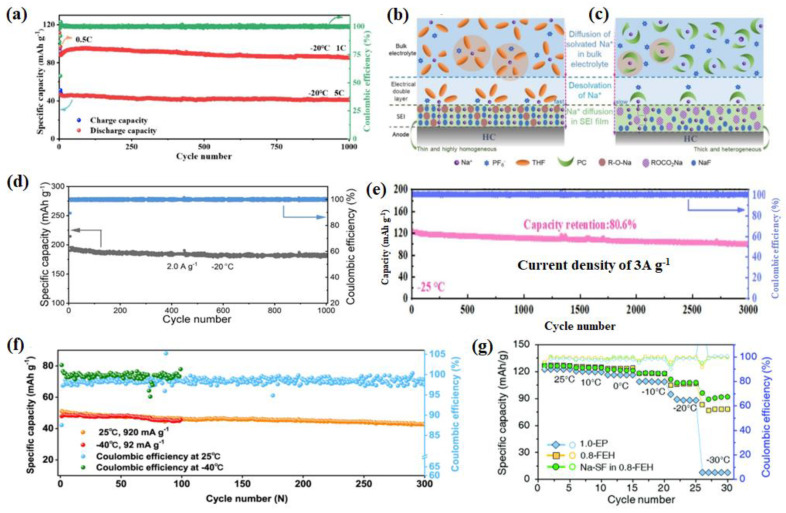
(**a**) Long-term cycles at −20 °C in 1 C and 5 C of SMBs. Reproduced with permission from ref. [139]. Copyright 2022 Elsevier B.V. Schematic diagrams of Na^+^ migration in (**b**) THF and (**c**) PC-based electrolytes and SEI at −20 °C. (**d**) Long-term cycling performance in 1 M NaPF_6_/THF. Reproduced with permission from ref. [140]. Copyright 2022 Wiley-VCH GmbH. (**e**) The cycle performance of defective HT-NW at 1 A g^−1^ and 3 A g^−1^ in 1.0 M NaCF_3_SO_3_ diglyme at −25 °C. Reproduced with permission from ref. [141]. Copyright 2021 Elsevier B.V. (**f**) Cycling performance at a rate of 920 mA g^−1^ at 25 °C and 92 mA g^−1^ at −40 °C. Reproduced with permission from ref. [143]. Copyright 2022 The Author(s). (**g**) Temperature-dependent performance of the Na/NVPOF cells in the various systems ranging from 25 °C to −30 °C at 0.2 C. Reproduced with permission from ref. [11]. Copyright 2021 Royal Society of Chemistry.

An ether-based electrolyte consisting of tetrahydrofuran as the main solvent was proposed in recent years. Yin et al. reported a novel electrolyte consisting of the main solvent (THF) and partial co-solvent (PC and PFPN), which is denoted as a TPP electrolyte [138]. Meanwhile, a thin and robust inorganic component-rich cathode electrolyte interface layer is elaborately introduced on the Na_2/3_Mn_2/3_Ni_1/3_O_2_ (MN) cathode by this tailored electrolyte, resulting in an excellent life cycle of the MN cathode (Figure 13d). The assembled Na_2/3_Mn_2/3_Ni_1/3_O_2_||Na cells with this TPP electrolyte exhibit a superior capacity retention of 94.1% after 100 cycles at a LT (−40 °C) (Figure 13e,f). Zhou et al. introduced a weak Na^+^ solvating co-solvent, tetrahydrofuran/1,2-Dime-thoxyethane (THF/DME), and the kinetic barrier for Na^+^ desolvation was significantly mitigated [139]. Furthermore, an anion-derived NaF-rich SEI film generated on the sodium electrode suppresses the dendrite growth and guarantees stable cycling of SMBs down to −60 °C. The full batteries deliver an initial capacity of 88.8 mAh g^−1^ at 1 C (Figure 14a), corresponding to 96.28% of the capacity produced at −20 °C after 1000 cycles. Furthermore, the batteries show a typical charge/discharge curve at −50 °C, indicating the superior adaptability to extreme temperature changes. Tang et al. proposed a strategy of electrode–electrolyte interfacial chemistry modulation, and in more detail, a new weakly solvating electrolyte (WSE) consisting of a cyclic ether (tetrahydrofuran, THF) as a solvent and NaPF_6_ as the electrolyte salt was proposed to optimize the interfacial properties of the anode/electrolyte [140]. As proven, weak ion solvation/desolvation of tetrahydrofuran greatly facilitates fast-ion diffusion at the SEI/electrolyte interface and homogeneous SEI with well-distributed NaF and organic components ensures fast Na^+^ diffusion through the SEI layer and a stable interface (Figure 14b,c). So that, even at −20 °C, a high capacity of 175 mAh g^−1^ (74% of its room-temperature capacity) can be maintained at 2 A g^−1^. This electrode retains 90% initial capacity after 1000 cycles (Figure 14d).

In electrochemical testing, ether-based electrolytes also have wide implications. For example, Sun et al. test the LT (−40 °C) electrochemical performance of flower-ball-like CuFeS_2_ embedded into the rGO nanosheet matrix (F-CuFeS_2_@rGO) with 1 M NaPF_6_ in a 2-MeTHF electrolyte [58]. Another study used hydrogen titanate nanowires (HT-NWs) as a model and revealed that layer structure regulation with oxygen defects could trigger HT-NWs to present unique Na^+^-solvent co-intercalation behavior in the ether-based electrolyte at −25 °C according to ex situ FTIR and XRD [141]. Benefiting from this, the defective HT-NW shows great superiority in cycle stability, maintaining a capacity retention of 80.6% after 4200 cycles at 1.0 A g^−1^ at −25 °C (Figure 14e). A novel desolvation-free sodium dual-ion battery (SDIB) has been proposed by using artificial graphite (AG) as the anode and polytriphenylamine (PTPAn) as the cathode. Such a SDIB operated with an ether-based electrolyte can intrinsically eliminate the sluggish desolvation process [142]. The desolvation-free mechanism endows the battery with 61% of its room-temperature capacity at an ultra-low temperature of −70 °C. Shi et al. used 1 mol L^−1^ NaPF_6_ dissolved in 100% diglyme as the electrolyte when testing the electrochemical performance (especially at LT) of a P_2_-type Na_0.78_Ni_0.31_Mn_0.67_Nb_0.02_O_2_ (P_2_-NaMNNb) cathode [143] (Figure 14f). In conclusion, ether-based electrolytes have advantages like a low melting point and low viscosity. They naturally perform well at LTs. Due to their low ability to dissolve and share solvents, ethers show excellent dynamic performance at both RT and LTs. The modification strategies of ether-based electrolytes primarily focus on enhancing their dynamic performance and adapting to LT environments. By reducing solvation energy, optimizing intercalation capabilities, and forming stable interfaces, ether-based electrolytes effectively improve sodium ion transport efficiency and cycling stability at LTs.

#### 2.3.3. Other Electrolytes

Researchers have recently discovered that electrolytes combining specific esters and ethers can exhibit excellent performance at LTs. For example, Zheng et al. tailored a Na-compatible electrolyte possessing weak Na^+^ solvation, consisting of 0.8 M NaPF_6_ in FEC/EMC/HFE (3:3:4 by vol) (denoted as 0.8-FEH); among them, the non-solvating, highly fluorinated HFE (Tm = −94 °C) further weakens the electrolyte affinity with Na^+^, accompanied by reduced electrolyte viscosity and flammability [11]. In the full battery test, under the combined action of the three components in the electrolyte, the system showed very surprising performance under LTs (Figure 14g). An insisting research hot topic emerges in solid electrolytes, emboldened by their qualities of unwavering thermal stability, a boundless electrochemical window, an enduring life cycle, and superb mechanical power. Solid electrolytes, of which many kinds have been reported, including ceramic-based, sulfide-based, and polymer electrolytes [144,145,146], in their steadfast natures, usually sidestep the quandary of phase transitions in the microtherm. The current research, under LT conditions, primarily focuses on the exploration of solid polymer electrolytes and the revelation of inorganic solid electrolytes [147]. In the lower temperature range, compared to solid polymer electrolytes, inorganic solid electrolytes have higher ionic conductivity, which is of great significance for LT solid SIBs [148].

## 3. Conclusions and Perspective

Over the past decade, SIBs have advanced rapidly, particularly for large-scale energy storage where other secondary batteries face challenges. To enhance their LT performance, extensive research has been conducted. Regarding electrode materials, we have systematically explored the LT properties of various anode materials based on different energy storage mechanisms. Notably, titanium-based insert anodes and polyanionic cathodes exhibit strong structural stability and compatibility with organic carbonate electrolytes that include functional additives. This makes them promising candidates for developing effective SIBs suitable for LT conditions. In terms of electrolytes, our discussion has focused on the recent progress of ester-based and ether-based electrolytes, offering potential pathways for future advancements in LT SIBs. Looking ahead, we anticipate promising developments in the field of LT SIBs.

(1) Innovative materials: New materials can revolutionize battery technology, especially SIBs. The advancement of batteries heavily relies on material development. For instance, materials with lots of active sites and good mass transfer can ensure fast reactions, high capacity, and long-lasting performance. Precisely arranging materials at a microscopic or even mesoscale using “precision chemistry” might enhance ion and electron movement in LT conditions. Ideally, materials with minimal energy barriers for ion and electron transfer could be universally applicable in such environments.
(2) More suitable electrolytes: Traditional electrolytes usually use ester-based solvents and have been extensively studied in LIBs and SIBs. In contrast, emerging electrolytes based on ether solvents lack a comprehensive understanding of their fundamental properties and interactions with lithium/sodium ions and solvated structures. In-depth basic research on ether solvents, especially their relationship with sodium ions and various salts at LTs, including the effects of different solvents and salts on ion transport performance, electrochemical stability, and thermal stability, can provide valuable insights to guide the development of new LT electrolytes for SIBs.
(3) Development of theoretical mechanisms: In recent decades, there have been advancements in new energy technologies, but our scientific understanding has not deepened much. While energy storage materials are progressing and new ideas are emerging, electrochemistry itself has not seen much conceptual innovation. Understanding the electrode and electrolyte interface, along with the dynamic nature of batteries, remains complex. A unified theory for electrochemistry at the system level is still lacking. Looking ahead, integrating computer technology may help establish more innovative theoretical frameworks.
(4) Around safety: Solid electrolytes are promising for enhancing the safety and stability of sodium metal batteries (SMBs) by avoiding issues like leaks and flammability seen with liquid electrolytes. Future research will likely concentrate on boosting the electrical conductivity and strength of solid electrolytes to better match SMB requirements. Advanced techniques for characterization will be crucial for understanding how these cells work, especially at the nanoscale and during operation. This exploration will provide valuable insights for subsequent optimization efforts.
